# Diagnostic validity of different cephalometric analyses for assessment of the sagittal skeletal pattern

**DOI:** 10.1590/2177-6709.23.5.075-081.oar

**Published:** 2018

**Authors:** Maheen Ahmed, Attiya Shaikh, Mubassar Fida

**Affiliations:** 1 Bakhtawer Amin Medical and Dental College, Dental Section, Department of Orthodontics (Multan, Pakistan).; 2 Liaquat College of Medicine and Dentistry (Karachi, Pakistan).; 3 The Aga Khan University, Department of Surgery, Section of Dentistry (Karachi, Pakistan).

**Keywords:** Diagnosis, Cephalometry, Reliability, Validity

## Abstract

**Introduction:**

Numerous cephalometric analyses have been proposed to diagnose the sagittal discrepancy of the craniofacial structures.

**Objective:**

This study aimed at evaluating the reliability and validity of different skeletal analyses for the identification of sagittal skeletal pattern.

**Methods:**

A total of 146 subjects (males = 77; females = 69; mean age = 23.6 ± 4.6 years) were included. The ANB angle, Wits appraisal, Beta angle, AB plane angle, Downs angle of convexity and W angle were used to assess the anteroposterior skeletal pattern on lateral cephalograms. The sample was classified into Class I, II and III groups as determined by the diagnostic results of majority of the parameters. The validity and reliability of the aforementioned analyses were determined using Kappa statistics, sensitivity and positive predictive value (PPV).

**Results:**

A substantial agreement was present between ANB angle and the diagnosis made by the final group (k = 0.802). In the Class I group, Downs angle of convexity showed the highest sensitivity (0.968), whereas ANB showed the highest PPV (0.910). In the Class II group, ANB angle showed the highest sensitivity (0.928) and PPV (0.951). In the Class III group, the ANB angle, the Wits appraisal and the Beta angle showed the highest sensitivity (0.902), whereas the Downs angle of convexity and the ANB angle showed the highest PPV (1.00).

**Conclusion:**

The ANB angle was found to be the most valid and reliable indicator in all sagittal groups. Downs angle of convexity, Wits appraisal and Beta angle may be used as valid indicators to assess the Class III sagittal pattern.

## Resumo

**Introdução:**

numerosas análises cefalométricas foram propostas para diagnosticar a discrepância sagital das estruturas craniofaciais.

**Objetivo:**

este estudo teve como objetivo avaliar a confiabilidade e validade de diferentes análises esqueléticas para a identificação do padrão esquelético sagital.

**Métodos:**

foram incluídos 146 indivíduos (homens = 77; mulheres = 69; idade média = 23,6 ± 4,6 anos). O ângulo ANB, a avaliação de Wits, o ângulo Beta, o ângulo do plano AB, o ângulo de convexidade de Downs e o ângulo W foram utilizados para avaliar o padrão esquelético anteroposterior em cefalogramas laterais. A amostra foi classificada nos grupos Classe I, II e III, conforme os resultados diagnósticos da maioria dos parâmetros. A validade e a confiabilidade das análises acima mencionadas foram determinadas usando estatísticas Kappa, sensibilidade e valor preditivo positivo (VPP).

**Resultados:**

foi encontrada uma concordância significativa entre o ângulo ANB e o diagnóstico feito pelo grupo final (k = 0,802). No grupo Classe I, o ângulo de convexidade de Downs mostrou a maior sensibilidade (0,968), enquanto o ANB apresentou o maior VPP (0,910). No grupo Classe II, o ângulo ANB mostrou a maior sensibilidade (0,928) e o maior VPP (0,951). No grupo Classe III, o ângulo ANB, a avaliação de Wits e o ângulo Beta apresentaram a maior sensibilidade (0,902), enquanto o ângulo de convexidade de Downs e o ângulo ANB apresentaram o maior VPP (1,00).

**Conclusão:**

o ângulo ANB foi considerado o indicador mais válido e confiável em todos os grupos sagitais. O ângulo de convexidade de Downs, a avaliação de Wits e o ângulo Beta podem ser usados como indicadores válidos para avaliar o padrão sagital de Classe III.

## INTRODUCTION

Variations in the normal craniofacial development in sagittal, vertical or transverse planes may result in different malocclusions.^1^ However, malocclusions in the sagittal plane have major esthetic, psychological and functional implications and are usually on top of the orthodontic problem list.^2^ ^,^ ^3^ A sagittal skeletal malocclusion may result from discrepancies in maxillary or mandibular growth. A more anteriorly positioned mandible with respect to the maxilla may result in a prognathic or concave profile; whereas, a relatively anteriorly positioned maxilla as compared to the mandible results in a retrognathic or convex profile. The skeletal discrepancies in the sagittal plane are best evaluated on radiographs in which both the morphology of different skeletal structures and their relationship to the surrounding tissues can be accurately assessed. Standardized lateral cephalogram has established itself as the classical tool to diagnose the sagittal discrepancies in the skeletal, dental and soft tissues.^4^

After the standardization of the cephalogram by Broadbent,^5^ the diagnosis of the anteroposterior skeletal problems has become a straightforward process. Various cephalometric analyses have been proposed for the evaluation of the sagittal skeletal discrepancies. Downs^6^ described the AB plane angle and Downs angle of convexity to assess the anteroposterior jaw dysplasia. In 1953, Riedel^7^ introduced the ANB angle, which was later popularized by Steiner.^8^ Studies have indicated that these angular measurements are sensitive to small changes in the position of nasion and sella turcica, length of the anterior cranial base and the vertical growth pattern.^9^ ^,^ ^10^ To overcome this limitation, Jacobson^10^ proposed the Wits appraisal, which employed the occlusal plane as the reference. However, the reproducibility and reliability of Wits appraisal has been questioned due to the variations in inclination and difficulties in identification of the functional occlusal plane.^11^ Hence, several other parameters have been and are still being introduced to overcome the shortcomings of the existing cephalometric analyses for an accurate diagnosis of sagittal discrepancies. Recently, the Beta angle and W angle have been proposed to evaluate the anteroposterior jaw dysplasia, but their diagnostic performance and validity have not yet been investigated.^12^ ^,^ ^13^

In the past, multiple researchers have correlated various cephalometric analyses for assessing anteroposterior jaw disrepancy.^14^ ^-^ ^18^ Ahmed et al^19^ reported the diagnostic accuracy of various cephalometric skeletal parameters for assessing the skeletal facial vertical pattern. However, to our knowledge, no such study has evaluated the reliability of anteroposterior skeletal dysplasia parameters. This has resulted in numerous parameters that need to be analyzed during cephalometric analysis, which is not only time-consuming, but sometimes may also provide conflicting results. Thus, this study aimed to identify the skeletal parameters that more accurately identified the sagittal skeletal pattern of an individual - since preference may be given to those analyses which are precise, consistent and reliable. This may not only improve the efficiency of the treatment planning process, but may also establish a reliable criteria for the classification of subjects into different sagittal malocclusion groups for research purposes. 

## MATERIAL AND METHODS

Data was collected retrospectively from the dental records of patients attended at the dental clinics of the authors. The sample size was calculated using the OpenEpi software (version 3.0) based on the findings of Gul-e-Erum and Fida.^16^ The alpha was taken as 0.05 and power of the study as 80% to calculate the sample size. Results have proposed a sample size with a minimum of 38 subjects in each group. As the subjects were divided into three groups based on vertical facial pattern, a minimum of 114 subjects were required. However, to increase the power of the study, a maximum number of subjects were included. A total of 198 subjects aged between 18 and 35 years (99 males and 99 females; mean age = 23.6 ± 4.6 years), having good quality lateral cephalograms were included. Patients with previous history of any orthodontic treatment, growth disturbance or facial trauma were excluded. Since variations in vertical growth pattern may be a confounding factor, only subjects with normal vertical growth pattern were included. This was determined when all the three vertical dysplasia parameters - FMA, SN-GoGn and PFH-TAFH - indicated a normodivergent growth pattern.^9^

**Figure 1:**
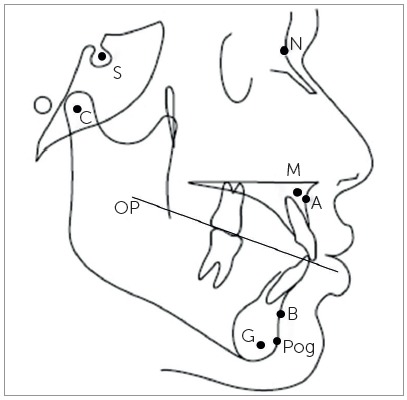
Cephalometric landmarks and the occlusal plane.

**Figure 2:**
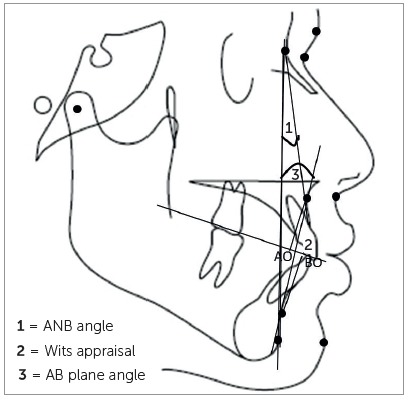
Cephalometric parameters.

**Figure 3:**
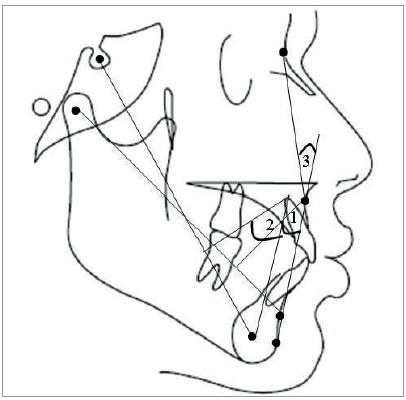
Cephalometric parameters.

The patients’ pretreatment lateral cephalogram taken in natural head position were used to determine the anteroposterior skeletal jaw discrepancy. The cephalograms were manually traced by the main investigator, the skeletal landmarks were identified and the following parameters were measured, as follows (Fig 1, 2, 3): 

1 = Beta angle: The angle formed by A-CB and AB line (normal range = 27° to 35°).

2 = W angle: The angle between the perpendicular line from point M to S-G line and the M-G line (normal range = 51° to 55°).

3 = Downs angle of convexity: The angle between N-point A and point A-Pog (normal range = -8.5° to 10°).

» ANB angle: the angle formed by point A, Nasion and point B (normal range = 0^o^ to 4^o^).^8^
» Wits appraisal: the linear distance between AO and BO (perpendicular drawn from point A and B on to functional occlusal plane) (normal range = -1mm to +1mm).^10^
» AB plane angle: the angle formed by AB plane and N-pog line (normal range = - 9^o^ to 0^o^).^6^
» Beta angle: the angle formed by A-CB and AB lines (normal range = 27^o^ to 35^o^).^12^
» W angle: the angle between the perpendicular line from point M to S-G line and the M-G line (normal range = 51^o^ to 55^o^).^13^
» Downs angle of convexity: the angle between N-point A and point A-Pog (normal range = -8.5^o^ to 10^o^).^6^

The norms of each skeletal analysis as established in literature were used to classify subjects as Class I, Class II and Class III.^6^ ^,^ ^8^ ^,^ ^10^ ^,^ ^12^ ^,^ ^13^ Fifty subjects were excluded from the study as they were found to have a similar sagittal skeletal pattern as determined by all the parameters. Each of the remaining 146 subjects (males = 77; females = 69) had at least one parameter giving conflicting diagnosis of the sagittal skeletal pattern. The final diagnosis of the anteroposterior growth pattern of the remaining subjects was based on the results of the majority of the analyses. This enabled to divide the remaining subjects into Class I, Class II and Class III anteroposterior groups. The final classification of the subjects resulted in the following groups:

» Class I: n = 63.
» Class II: n = 42.
» Class III: n = 41.

‘Correctly diagnosed cases’ were labeled when a specific skeletal analysis in a subject matched the final diagnosis. These were then used to assess the diagnostic accuracy of each parameter.

Thirty cephalograms were retraced and randomly reanalyzed by the main investigator. The errors were calculated according to Dahlberg’s formula^20^ and the coefficient of reliability (ICC). The Dahlberg’s error ranged from 0.103 to 0.890, while the results for the ICC showed a high correlation between the two sets of readings (Table 1). 

**Table 1:** Intraclass Correlation Coefficient.

Measurements	1^st^ reading (n=30)	2^nd^ reading (n=30)	ICC	Dahlberg’s calculations
ANB	1.67 ± 4.81	1.87 ± 4.96	0.987	0.646
Wits appraisal	-1.48 ± 5.46	-1.48 ± 5.59	0.943	0.103
Beta angle	34.13 ± 8.82	34.40 ± 8.92	0.989	0.245
AB plane angle	-2.80 ± 7.76	-2.87 ± 7.93	0.992	0.480
Downs angle of convexity	1.63 ± 10.29	1.67 ± 10.38	0.993	0.560
W angle	54.40 ± 5.82	54.67 ± 5.97	0.989	0.890

ICC: Intraclass correlation coefficient. n=30.

SPSS for Windows (version 20.0, SPSS Inc. Chicago) was used for data analysis. The anteroposterior skeletal analyses were evaluated using the Pearson’s correlation. Kappa statistics were applied to assess the level of agreement between the skeletal analyses and the final diagnosis made from the ‘correctly diagnosed cases’. The validity in terms of sensitivity and Positive predictive value (PPV) were determined from the two by two tables. A p-value < 0.05 was taken as statistically significant.

## RESULTS

The sample comprised 146 subjects (69 females, mean age = 20.67 ± 4.8 years; 77 males, mean age = 21.98 ± 4.8 years). The means and standard deviations of each parameter in all three sagittal malocclusions are shown in Table 2.

**Table 2:** Mean value of cephalometric parameters.

Parameter	Class I n = 63 mean ± SD	Class II n = 42 mean ± SD	Class III n = 41 mean ± SD
ANB	1.30 ± 1.76	6.45 ± 1.31	-2.17 ± 2.52
Wits appraisal	0.389 ± 3.01	4.36 ± 3.78	-6.30 ± 5.24
Beta angle	32.49 ± 5.43	26.31 ± 4.03	43.54 ± 4.75
AB plane angle	-5.14 ± 3.5	-10.48 ± 4.12	3.20 ± 3.51
Downs angle of convexity	4.00 ± 3.94	11.29 ± 3.65	-3.66 ± 3.12
W angle	53.83 ± 3.94	49.45 ± 2.52	58.46 ± 2.54

Correlation between the different skeletal analyses was determined using Pearson’s correlation. A strong correlation was present between the ANB angle and Wits appraisal (r = 0.831, *p* < 0.01), and ANB angle and Downs angle of convexity(r = 0.823, *p* < 0.01) (Table 3).

**Table 3:** Correlation among different skeletal analyses to assess sagittal growth pattern.

	ANB	Wits Appraisal	Beta Angle	AB Plane Angle	Down’s Angle of Convexity	W Angle
ANB	1	0.831**	-0.775**	-0.783**	0.823**	-0.704**
Wits appraisal		1	-0.730**	-0.625**	0.634**	-0.654**
Beta angle			1	-0.694**	-0.680**	0.636**
AB plane angle				1	-0.792**	0.568**
Downs angle of convexity					1	-0.678**
W angle						1

n = 146. Pearson correlation: weak correlation (± 0.01 < r < ± 0.5); moderate correlation (± 0.5 < r < ± 0.8); strong correlation (± 0.8 < r < ± 1)

*p < 0.05; ** p < 0.01.

Kappa statistics assessed the agreement among diagnostic criteria of different cephalometric analyses. A substantial agreement was present between the ANB angle and the final group (k = 0.802, *p*< 0.01) (Table 4). 

**Table 4:** Assessment of agreement among diagnostic criteria of skeletal analyses.

Parameter	Class I	Class II	Class III	Kappa	P-value
	n = 63	n = 42	n = 41	n =146	
ANB	56	53	37	0.802**	0.000
Wits appraisal	31	64	51	0.489**	0.000
Beta angle	71	23	52	0.511**	0.001
AB plane angle	70	35	41	0.724**	0.000
Downs angle of convexity	112	32	2	0.397**	0.000
W Angle	60	36	50	0.530**	0.0401

n = 146; Kappa Statistics. *p < 0.05; ** p < 0.01

PPV and sensitivity of each diagnostic parameter were also calculated for each group separately. In the Class I group, Downs angle of convexity showed the highest sensitivity (0.968), whereas the ANB angle showed the highest PPV (0.910). In the Class II group, the ANB angle showed the highest sensitivity (0.928) as well as the highest PPV (0. 951). In the Class III group, the ANB angle, Wits appraisal and the Beta angle showed the highest sensitivity (0.902), whereas the Downs angle of convexity and the ANB angle showed the highest PPV (1.00) (Table 5).

**Table 5:** Assessment of positive predictive value and sensitivity of various parameters to assess sagittal discrepancy.

Parameter	Class I (n = 63)			Class II (n = 42)			Class III (n = 41)		
	Correctly diagnosed cases	Positive Predictive value	Sensitivity	Correctly diagnosed cases	Positive Predictive value	Sensitivity	Correctly diagnosed cases	Positive Predictive value	Sensitivity
ANB	51	0.910	0.809	39	0.951	0.928	37	1.00	0.902
Wits appraisal	22	0.710	0.349	36	0.563	0.857	37	0.740	0.902
Beta angle	44	0.619	0.698	19	0.826	0.452	37	0.711	0.902
AB plane angle	54	0.771	0.857	30	0.857	0.714	36	0.878	0.878
Downs angle of convexity	61	0.545	0.968	30	0.937	0.714	2	1.00	0.488
W Angle	39	0.650	0.619	27	0.750	0.642	35	0.700	0.853

n = 146.

## DISCUSSION

In Orthodontics, great importance has been advocated to the cephalometric assessment of the jaw relationship in the sagittal plane. Since the advent of lateral cephalometry by Broadbent^5^, various analyses have been proposed to assess the anteroposterior jaw relationship.^6^ ^,^ ^8^ ^,^ ^10^ ^-^ ^12^ In borderline cases, several skeletal analyses may show conflicting results, and a clear cut diagnosis regarding the sagittal skeletal pattern is not possible. This study aimed to concise the process of diagnosis to minimal skeletal parameters by evaluating the diagnostic accuracy of the most commonly used analyses. 

A ‘final diagnosis’ of the anteroposterior skeletal pattern was based on the results of majority of the parameters. This ‘final diagnosis’ was then treated as gold standard. The diagnostic accuracy of the included skeletal parameters was then compared using kappa statistics, PPV and sensitivity. 

In the present study, all the analyses showed significant correlation with each other. A strong positive correlation was present between the Wits appraisal and ANB angle (r = 0.831), and the ANB angle and Downs angle of convexity (r = 0.823). Ishikawa et al^14^ reported a strong correlation between AB plane angle and Downs angle of convexity (r = -0.86), AB Plane angle and the ANB angle (r = -0.95), and the ANB angle and Downs angle of convexity (r = 0.97). The variations in results may be due to differences in sample size and inclusion of only Class I subjects. In another study by Gul-e-Erum and Fida,^16^ a strong correlation was reported between AB plane angle and ANB (r = 0.749). The present study reported similar findings.

The strength of the correlation does not indicate whether the specific parameter can precisely diagnose the skeletal anteroposterior parameter. Hence, in the present study, to compare the diagnostic agreement between various skeletal analyses and the final diagnosis, Kappa statistics were applied. A substantial agreement was present between the final group and ANB angle (k = 0.802). The Kappa statistic explains the variation in diagnosis that may occur simply as a result of chance.^21^ Hence, the ANB angle was found to be the most reliable indicator in precisely assessing the sagittal skeletal pattern of a patient.

It is of prime importance for an analysis to diagnose a certain parameter with consistency and accuracy. Hence the sensitivity of each parameter was determined to validate their diagnostic accuracy. Downs angle of convexity showed the highest sensitivity in the Class I group (0.968), whereas ANB angle was found to be the most sensitive parameter in Class II group (0.928). In the Class III group, ANB angle, Wits appraisal and Beta angle (0.902) were found to have the highest sensitivity in evaluating the sagittal growth pattern. Thus in evaluating the sagittal growth pattern with precision in an individual , Downs angle of convexity and the ANB angle may be used as valid indicators in Class I and Class II subjects. In the Class III group, ANB angle, Wits appraisal and Beta angle may be used to accurately assess the sagittal growth pattern of an individual.

In the present study, to confirm whether a certain parameter can accurately depict the skeletal pattern, the positive predictive values (PPV) were also calculated for each group separately. The ANB angle yielded the highest PPV in Class I (0.910) and Class II (0.951) sagittal groups. In the Class III sagittal group, ANB angle and Downs angle of convexity showed the highest PPV (1.00). Thus, the ANB angle in all three sagittal groups has a high probability for correctly diagnosing the anteroposterior jaw dysplasia. In addition, if Downs angle of convexity is indicating a Class III jaw relationship in a particular individual, then it is highly likely to be true and may not need to be verified by other analyses.

A number of studies have indicated that the hyperdivergent or hypodivergent vertical growth pattern may affect the sagittal jaw relationship.^9^ ^,^ ^10^ This may reduce the accuracy and precision in evaluating the diagnostic accuracy of the existing sagittal jaw dysplasia parameters. In our study, the ANB angle was seen to accurately determine the anteroposterior jaw dysplasia in normodivergent subjects. 

Hence, the sagittal analyses for evaluating the skeletal discrepancy may be limited to fewer analyses. These analyses showed higher diagnostic performance, as compared to other parameters. This may result in an accurate and time-saving diagnosis, thus increasing the efficiency of the treatment planning process. Moreover, the present study also provides reliable criteria for the classification of subjects for various research purposes.

## CONCLUSION

All the skeletal parameters showed a significant correlation with each other. The ANB angle was found to be the most reliable and valid indicator in assessing the anteroposterior jaw relationship in all sagittal groups. Hence, it may be used to precisely and accurately assess the sagittal jaw discrepancy. In addition, Downs angle of convexity, Wits appraisal and Beta angle may be used as valid indicators to assess the Class III sagittal growth pattern. 
